# Effectiveness of the Austrian disease-management-programme for type 2 diabetes: study protocol of a cluster-randomized controlled trial

**DOI:** 10.1186/1745-6215-9-38

**Published:** 2008-06-19

**Authors:** Andreas C Sönnichsen, Andreas Rinnerberger, Maria G Url, Henrike Winkler, Peter Kowatsch, Gert Klima, Bernhard Fürthauer, Raimund Weitgasser

**Affiliations:** 1 Institute of General Practice, Family Medicine and Preventive Medicine, Paracelsus Medical University, Strubergasse 21, 5020 Salzburg, Austria; 2 Workinggroup for Preventive Medicine, Salzburg (AVOS) Elisabethstrasse 2, 5020 Salzburg, Austria; 3 Styrian Public Health Insurance, Josef-Pongratz-Platz 1, 8011 Graz, Austria; 4 Austrian Association of General Practice (ÖGAM), Alser Strasse, 1090 Vienna, Austria; 5 Medical Department I, University Hospital, Paracelsus Medical University, Müllner Hauptstrasse 48, 5020 Salzburg, Austria; 6 Austrian Diabetes Association (ÖDG), Währingerstrasse 76/13, 1090 Vienna, Austria

## Abstract

**Background:**

Due to its rising prevalence type 2 diabetes plays an important role concerning population health in Austria and other western countries. In various studies deficiencies in the care of diabetic patients have been revealed. These deficiencies may be overcome by disease-management-programmes (DMPs), but international experience shows that the effectiveness of DMPs is inconsistent. In particular large programmes designed by state-affiliated public health insurances have not been evaluated in randomized controlled trials (RCTs). We are therefore conducting a large scale RCT of the Austrian DMP for type 2 diabetic patients in the province of Salzburg to evaluate the programme regarding its effects on metabolic control, guideline adherent care and the quality of life of diabetic patients.

**Methods/Design:**

The study is open for participation to all GPs and internists in the province of Salzburg. Physicians are randomized before recruitment of patients with the districts of Salzburg as clusters of randomisation. A total of over 1200 patients with type 2 diabetes will then be recruited. In the intervention group the DMP is applied for one year. Controls receive usual care. Endpoints are a decrease in HbA1c in the intervention group > 0,5% compared to controls, a higher percentage of patients with required diagnostic measures according to guidelines, improved cardiovascular risk profile and higher quality of life scores within one year.

**Current status of the study:**

98 Physicians agreed to participate in the study. 96 of them recruited 1494 patients, 654 in the intervention and 840 in the control group.

**Trail Registration:**

This trial has been registered with Current Controlled Trials Ltd. (ISRCTN27414162).

## Background

The prevalence of type 2 diabetes is rising worldwide [1]. In Austria about 3,5 – 5% of the population are affected and it has been shown that diabetes care is insufficient [2]. The socioeconomic impact of the disease is alarming and it is necessary to improve its prevention and control. Effective screening for early detection and structured care according to current guidelines are the most promising strategies to avoid diabetic complications. One of the concepts to assure optimized care is the design of disease management programmes comprising physician training in guideline-adherent therapy and patient education as well as reminders and continuous feedback. Unfortunately disease management programmes don't seem to guarantee optimum care. As has been shown in Germany, a nation-wide implementation of DMPs by mandatory public health insurances may rather lead to increasing bureaucracy than to an improvement in care. Therefore the programmes have been criticized widely [3]. Evaluation studies have not been carried out as randomized controlled trials, and have been restricted to small samples of highly motivated physicians. Thus, results so far are not sufficient to support a general implementation [4]. Randomized controlled trials that have been performed in various countries have never evaluated large programmes of state-affiliated public health insurances, and showed only limited success, especially regarding clinically relevant endpoints. DMPs may – depending on the programme – improve surrogate parameters like HbA1c, but didn't show any effect on cardiovascular morbidity and mortality after six years of observation [5].

In a systematic review the authors conclude that the majority of DMPs lead to an improvement in metabolic control, but this appears to be largely dependent on the individual programme and its particular design [6]. Thus, physician training leads to an improvement of metabolic control only in 38% of the studies included in the review, and to increased guideline adherence in 50% of the studies. Patient education was shown to be effective in 44% of the studies.

Another systematic review published in 2004 came up with similar results. Only in 24 out of 66 randomized controlled trials on the effectiveness of DMPs a significant improvement in patient care and metabolic control was demonstrated [7].

One systematic review achieved slightly better results but included non-controlled studies as well as studies performed in HMOs (Health Maintenance Organisations) and Community Clinics. The results can hardly be transferred to single GP surgeries [8].

The effectiveness of patient education has been explicitly evaluated in a Cochrane-Review. The authors conclude with caution that patient education in groups may tend to improve metabolic control. 10 out of the 11 studies included in the review are considered to have serious methodological deficits [9]. Among these the study of Pieber et al. is also criticised. The development of the DMP of the Austrian Public Health Insurance has been partly based on this study. It is characterised by non-randomized controls, a short observation period and a small number of highly motivated surgeries [10].

All these reviews show clearly that there are large discrepancies and variations from study to study regarding the effectiveness of a DMP. One of the explanations may be that there are important differences in the design, making each DMP a unique intervention in a unique setting that may not be comparable to other DMPs even though the basic elements (physician training, patient education, reminders etc.) remain the same. We therefore believe that a newly designed DMP must be evaluated in its particular setting before general implementation can be recommended. This is especially true for large public programmes that not only impose additional work on the surgeries involved but also cause tremendous cost to the health care system.

The prerequisites of summative evaluation of public health interventions have been described in the context of international expertise and comprise the performance of a randomized controlled trial prior or at least parallel to their general implementation [11]. In several instances, i.e. in the implementation process of DMPs by public health insurances in Germany this rigorous evaluation has not been performed, leading to persistent criticism and scepticism regarding the effectiveness of these programmes [12]. Now a summative and controlled evaluation is not possible anymore because the programmes have been implemented all over the country.

In Austria we are currently facing the plan of a nationwide implementation of a DMP for type 2 diabetes. To avoid the lack of a summative evaluation as presently missing in Germany we suggested to the public health insurance and health authorities of Salzburg to implement the DMP starting with a RCT.

## Objectives of the study

The study focuses on the following aspects of effectiveness of the DMP for type 2 diabetic patients (called „Therapie aktiv“) designed by the Austrian public health insurance:

• Does metabolic control improve in type 2 diabetics participating in the DMP „Therapie aktiv“ compared to controls receiving usual care after one year of observation?

• Is guideline-adherent care regarding metabolic control and early detection of diabetic complications improved in DMP-patients compared to controls receiving usual care?

• Is guideline-adherent therapy of type 2 diabetes and its complications improved in DMP-patients compared to controls receiving usual care?

• Is guideline-adherent therapy of cardiovascular risk factors improved in DMP-patients compared to controls receiving usual care?

The primary objective of the study is to show that the Austrian DMP for diabetes mellitus type 2 improves metabolic control of patients with diabetes. This improvement must exceed a decrease of HbA1c of 0.5% between the beginning and the end of the study in the intervention group compared to the controls (see endpoints).

Secondary objectives are to demonstrate that the Austrian DMP leads to an improvement of guideline adherent treatment and an increased frequency of the recommended preventive examinations regarding diabetic complications (i.e. frequency of eye examinations, foot examinations, testing for microalbuminuria, serum creatinine and plasma lipids). Another secondary objective of the study is the calculation of the effect of the DMP on economic issues (i.e. prospective hospital admissions and economic burden due to diabetic complications).

## Methods/Design

### Study Design

The study is performed as a cluster-randomized controlled trial of a complex intervention with an observation time of one year. Randomization-clusters are the districts of the province of Salzburg because randomization is neither feasible at the level of the patient nor at the level of the GP. Randomization at the level of the patient leads to the problem that a single GP would have to treat some patients according to usual care and others according to the DMP which is hardly possible because of "contamination" effects. Also, randomization at the level of the GP appears not to be advisable, because this would lead to contamination effects especially in rural areas, where neighbouring GPs have overlapping patient groups. Patients knowing one another and belonging one to the intervention and the other to the control group, may even switch their physician.

### Setting and Study Population

The study takes place in the province of Salzburg, consisting of Salzburg city with about 150.000 inhabitants and five districts (suburban and rural areas including a few larger villages with a total of about 350.000 inhabitants. The prevalence of type 2 diabetes in this population is estimated to be about 2.5 to 3%.

### Recruitment and Randomization

#### Recruitment of the surgeries

Participation in the study is offered to all physicians who are eligible to participate in the DMP, i.e. all GPs and specialists in internal medicine with their own surgery and a contract with the public health insurance (252 GPs and 23 specialists in internal medicine). We inform all of the 275 physicians in writing about the study and its objectives and ask them to respond by letter or fax to declare their willingness to participate. Furthermore, we offer additional information by phone. All physicians are informed explicitly that the study will be a randomized controlled trial, and that randomization will only take place after a commitment to participate has been made. Therefore, at the time of signing up the physicians do not know which group – intervention or control – they will belong to.

#### Randomisation

Randomisation takes place at the level of the districts after completion of physician recruitment. The city of Salzburg as the only urban region of the province is divided into two study districts (one being to the right, the other to the left of the Salzach, the river that divides the city in nearly two halves). To assure even distribution of the districts regarding population characteristics and size the districts are randomized as matched pairs. Salzburg city to the right of the Salzach is matched with the city left of the Salzach, the district Pongau is matched with Pinzgau and Lungau (mostly mountainous regions with remote villages), and the district Flachgau is matched with Tennengau (both adjacent to Salzburg city with partially suburban and partially agricultural regions, more densely populated than the mountain districts). The randomization process is performed under the supervision of a committee comprising representatives of Paracelsus University, the Austrian public health insurance, the Working-group for Preventive Medicine, Salzburg (AVOS) and the healthcare department of the government of the province.

#### Recruitment of patients

All patients (> 18 years) with type 2 diabetes that fulfil the WHO/ADA-criteria for diabetes diagnosis are eligible to participate in the study. The physicians are encouraged to continuously recruit all patients with type 2 diabetes that enter the surgery during the recruitment period (from July 1^st ^to October 31^st ^of 2007). All patients willing to participate are included in the study after informed consent according to the declaration of Helsinki.

Exclusion criteria are dementia/psychiatric illness with inability to participate or to give informed consent, or known major consuming illness (i.e. advanced cancer).

### Intervention

In the intervention group the complex intervention of the DMP for type 2 diabetes as designed by the Austrian public health insurance ("Therapie aktiv") is applied, consisting of:

• a 10-hour training course for all physicians of the intervention group as designed by the Austrian Diabetes Association (ÖDG), the Austrian Medical College (Ärztekammer), and the Austrian Society for General Practice (ÖGAM). Physicians are trained by experienced GPs and specialists from the diabetes unit of the Medical Department I of the University Hospital Salzburg, and from the Institute of General Practice, Family Medicine and Prevention, both of Paracelsus Medical University (PMU)

• 9 hours of patient-education in 4 modules with a group size of 3 to 12 patients. Patient education is organized by the Workgroup for preventive medicine Salzburg (AVOS) according to the curriculum of the „Düsseldorfer Modell“ [13] and performed by especially trained physicians in their surgeries or in hospital out-patient clinics. The modules have been offered all over the province long before the implementation of the DMP (i.e. the study) but have not been used widely enough.

• documentation in a DMP-form at the beginning and at the end of the study year.

• structured interdisciplinary care as prescribed in the DMP according to the guidelines of the Austrian Diabetes Association (ÖDG).

• patient and physician reminders every three months sent out by the public health insurance to assure adherence to the schedule of prescribed diagnostic and therapeutic measures.

• agreement on therapeutic goals in a shared decision-making process of patient and physician.

### Control

In the control group the physicians perform the standard care as prescribed by current guidelines for the management of type 2 diabetes. Continuous medical education and patient education are allowed at the discretion of the particular physician or patient.

### Documentation and Monitoring

#### Baseline-Examination

At inclusion the following parameters are examined and recorded:

**• Laboratory**: Fasting and postprandial blood glucose, HbA1c, cholesterol, triglycerides, HDL-cholesterol

**• Anthropometric measurements**: Height and body weight, systolic and diastolic blood pressure

**• Case Report Form**: In the intervention group the DMP documentation form is used. To avoid bias, a simplified case report form is used in the control group. Some parameters (i.e. known diabetic complications) will be registered at the end of the study, see below).

#### Final examination

After one year baseline data will be recorded once again. In an additional case report form diabetic complications (present already at the beginning, and those that arose during the year), cardiovascular events, hospitalization, hypoglycaemias, diagnostic and therapeutic measures as well as therapeutic goals will be recorded (i.e. ophthalmological, neurological, podological, and nephrological examinations). Quality of life will be evaluated with the German version of the EQ-5 which has been used in the UKPDS (Version EQ5-D) [14].

### Endpoints

The primary endpoint of the trial will be the change in HbA1c from baseline to 12 months (final examination). Secondary outcomes will include a reduction in the amount of time spent in a hospital during the year of the study, a higher percentage of patients with guideline adherent diagnostic and therapeutic measures (i.e. eye examination, foot examination frequency) during the study, as well as improved control of cardiovascular risk and a higher EQ5-D-score in the intervention group at the end of the study.

The differences between the intervention group and the control group regarding primary and secondary endpoints will allow an estimate of the impact of the DMP on health economics.

### Data management and calculation of sample size

All data are recorded in the surgeries of the participating physicians and then transferred to the Institute of General Practice, Family Medicine and Prevention of the PMU for further processing and evaluation.

Sample size was calculated using the primary endpoint, i.e. the change in HbA1c from baseline to final examination at 12 months. Using an estimate of standard deviation for HbA1c change of 2%, a total of 504 patients (252 per arm) would be required to detect a difference of HbA1c of 0.5% (effect size, 0.25) with a power of 1-β = 80% using a two-sided two-sample t-test at a 0.05 significance level. Assuming an intracluster correlation coefficient of 0.05 and an average cluster size of 20, we estimated a design effect of D = 1 + (20-1) × 0.05 = 1.95. Thus, to have adequate power, the sample size had to be increased to 984 patients (492 per arm). Assuming a drop-out-rate of 20%, the sample size was adjusted to 615 patients per arm, or a total of 1230 patients.

### Statistical Analysis

All data will be analyzed on an intention to treat basis. The primary efficacy analysis will be to compare the change in the HbA1c from baseline to 12 months in the intervention and control groups. For all primary and secondary outcome variables, we will analyze differences between groups by using mixed models in SPSS 13.5 to allow for clustering. We will add all relevant baseline characteristics at patient level (age, sex, educational level, cardiovascular risk, clinical vascular disease, medication for primary and secondary prevention) as well as at cluster level if applicable as covariates to the models. Results of two-sided tests between study arms will be regarded significant at p < 0.05.

### Ethical issues and trial registration

This trial has been approved by the ethics committee of Salzburg, Austria, and has been registered with Current Controlled Trials Ltd. (ISRCTN27414162).

### Current status of the study

Recruitment of the surgeries took place from April 18^th ^to June 5^th ^2007. 98 Physicians declared themselves willing to participate in the study (88 GPs and 10 specialists in internal medicine) yielding a participation rate of 35.6% of all physicians eligible. Randomization took place on June 5^th ^2007. Salzburg city to the left of the Salzach river and the districts Pinzgau, Lungau and Tennengau were allocated to the intervention group (48 physicians), Salzburg city to the right of the Salzach river and the districts Flachgau and Pongau form the control group (50 physicians). The physicians of the intervention group received their DMP-training-courses in June and July of 2007. Recruitment of patients took place from July 1^st ^to October 31^st ^2007. Two surgeries dropped out and didn't recruit any patients. 1494 patients entered the study (654 in the intervention group and 840 in the control group). Baseline data are currently being processed.

## Discussion

Our study not only aims at a thorough summative evaluation of the Austrian DMP for type 2 diabetes, it also represents the first RCT of a DMP developed and implemented by a state-affiliated public health insurance. Such a study was proposed in Germany before the implementation of DMPs but for political reasons has never been carried out [3,12]. The current evaluation of the Austrian DMP avoids these deficiencies and will yield the necessary data to estimate effectiveness and economic effects of the programme. A major drawback of course is the study length. A study period of only one year will not be sufficient to show any effects on clinically relevant endpoints such as cardiovascular events, diabetic complications or mortality. This drawback, however, is encountered in many studies of therapeutic interventions which only investigate the effect on surrogate parameters like HbA1c. The UKPDS has shown the HbA1c to be a relevant surrogate for clinical endpoints in the long run [15,16]. Thus an improvement of HbA1c attributable to a DMP may be taken as the best available evidence for a clinically relevant effect. We therefore chose the HbA1c as the primary endpoint of our trial. HbA1c-associated effects on microvascular disease and health economics may be estimated from the results of the UKPDS. In a metaanalysis of the relationship between HbA1c and cardiovascular outcome a 1.18 fold increase of risk has been shown for a 1-percentage point increase of HbA1c [17]. Using this risk prediction in the comparison between intervention and control group the possible benefit regarding morbidity, mortality and economic factors pertaining to macrovascular disease may be cautiously estimated.

Another drawback of our RCT is the possibility of bias due to the cluster randomization and the lack of blinding. Of course, blinding of a complex intervention is not possible. Thus physicians in the intervention group may recruit different patients than those in the control group. Adjustments for these differences using multivariate testing will help to avoid and overcome this bias. As mentioned above, we chose randomization at the level of the district to avoid contamination. Nonetheless we prefer to perform the evaluation at the patient level because the benefit regarding clinical outcome is most important to the individual patient. The drawback of this approach is that cluster effects may be underestimated. Specific analysis of these effects and inclusion in the evaluation models will help to avoid this bias.

Another possible weakness may be the sample of participating physicians. Although the overall participation rate was quite high, the sample may not be representative for all GPs and internists in the country. It rather represents a subgroup more open to innovation and highly motivated to improve patient care. Nevertheless we feel that the sample may be quite representative for the physicians that will finally apply the DMP in everyday practice.

## Conclusion

If the DMP does not work in the study it will surely not work with the physicians that are less motivated than those under investigation. We therefore believe this trial to be a landmark in the evaluation of DMPs developed and implemented by public health insurance. Revealing positive results will not only allow an estimate of the DMP's impact on health economics, but will also lead to an increased acceptance of the DMP among GPs and thus improve diabetes care in the population. This may have a long-term effect on healthcare and health economics in Austria and in countries with similar public programmes. Furthermore the present programme can be optimized using the results of the RCT.

## List of abbreviations

ADA: American Diabetes Association; AVOS: Arbeitskreis Vorsorgemedizin Salzburg (Working-group for Preventive Medicine, Salzburg); DMP: disease management programme; GP: General practicioner; HMO: Health Maintenance Organisation; ICC: Intra-cluster-correlation coefficient; ÖDG: Österreichische Diabetes Gesellschaft (Austrian Diabetes Association); ÖGAM: Österreichische Gesellschaft für Allgemeinmedizin (Austrian Association of General Practice); PMU: Paracelsus Medical University; RCT: Randomized controlled trial; SAGES: Salzburger Gesundheitsfonds (Health Fund of the Province of Salzburg); SGKK: Salzburger Gebietskrankenkasse (Health Insurance of the Province of Salzburg); WHO: World Health Organization

## Competing interests

This study is partially financed by the public health insurance of Salzburg (SGKK) who may be interested in a positive study result. Smaller grants are provided by the Salzburg Savings Bank (Salzburger Sparkasse) and Roche Diagnostics Austria. Both may be interested in a positive study result for public relations reasons. The authors themselves, however, do not receive any reimbursement or financial benefits from these organisations.

## Authors' contributions

ACS developed the study idea, and drafted the study protocol as well as this manuscript, AR, MGU, HW, PK, GK, BF, and RW contributed equally to the development of the study protocol and to writing this manuscript, PK is in charge of the development and organization of patient education, GK has been in charge of the development of the DMP "Therapie aktiv", RW contributed to the development of the DMP, physician- and patient-education, and to the scientific evaluation as a specialist in type 2 diabetes.
